# IMPROVEMENT OF CAREGIVER MASTERY IS RELATED TO DECREASED ANXIETY AMONG PEOPLE LIVING WITH COGNITIVE IMPAIRMENT

**DOI:** 10.1093/geroni/igac059.2088

**Published:** 2022-12-20

**Authors:** Yeji Hwang, Miranda McPhillips, Liming Huang, G Adriana Perez, Nancy Hodgson

**Affiliations:** Drexel University, Philadelphia, Pennsylvania, United States; University of Pennsylvania, Philadelphia, Pennsylvania, United States; University of Pennsylvania, Philadelphia, Pennsylvania, United States; University of Pennsylvania, Philadelphia, Pennsylvania, United States; University of Pennsylvania, Philadelphia, Pennsylvania, United States

## Abstract

Anxiety is common and distressing in people living with cognitive impairment. High caregiver mastery, a positive view of one’s ability to provide care, is known to be protective against anxiety in people whom they are caring for. However, the longitudinal relationship between caregiver mastery and anxiety is unknown. Our objective was to examine whether improvement in caregiver mastery was related to decrease in anxiety in people living with cognitive impairment. This was a secondary data analyses using Healthy Patterns Clinical Trial (NCT03682185), an RCT of a home-based activity intervention designed to improve circadian rhythm disorders in people living with cognitive impairment. A total of 158 participants with cognitive impairment who provided data at both pretest (T1) and posttest (T2) were analyzed. Measures included Caregiver Mastery Scale and Neuropsychiatric Inventory. We used linear regression analyses to examine the relationship between changes in caregiver mastery and changes in anxiety. The sample was primarily female (66.7%), Black (63.1%), with mean age 73.3±8.4. The mean change of anxiety frequency (T2-T1) was -0.2±1.0; anxiety frequency decreased over time. The mean change of caregiver mastery (T2-T1) was 0.4± 3.0; caregiver mastery improved over time. After controlling for age, cognition, changes in sleep impairments, changes in depression, and intervention group assignment, improvement of caregiver mastery over time (B=-0.087, SE=0.035, t=-2.51, p=0.013) was related to decreased anxiety frequency over time (R2= 0.112, F=2.31, p= 0.039). Interventions to improve caregiver mastery may alleviate anxiety symptoms in people living with cognitive impairment. Additional research and practice implications will be discussed.

